# Telomere Biology and Thoracic Aortic Aneurysm

**DOI:** 10.3390/ijms19010003

**Published:** 2017-12-21

**Authors:** Thomas Aschacher, Olivia Salameh, Florian Enzmann, Barbara Messner, Michael Bergmann

**Affiliations:** 1 Cardiac Surgery Research Laboratory, Department of Surgery, Medical University of Vienna, Waehringer Guertel 18-20, 1090 Vienna, Austria; barbara.messner@meduniwien.ac.at; 2 Plastic and Reconstructive Surgery, Department of Surgery, Medical University of Vienna, Waehringer Guertel 18-20, 1090 Vienna, Austria; olivia.salameh@meduniwien.ac.at; 3 Department of Vascular and Endovascular Surgery, Paracelsius Medical University, Muellner Hauptstrasse 48, 5020 Salzburg, Austria; fkenzmann@gmail.com; 4 Surgical Research Laboratories, Department of Surgery, Medical University of Vienna, Waehringer Guertel 18-20, 1090 Vienna, Austria; michael.bergmann@meduniwien.ac.at; 5 Comprehensive Cancer Centre, 1090 Vienna, Austria

**Keywords:** telomeres, telomere maintenance mechanism, telomerase, aging, aortic aneurysm

## Abstract

Ascending aortic aneurysms are mostly asymptomatic and present a great risk of aortic dissection or perforation. Consequently, ascending aortic aneurysms are a source of lethality with increased age. Biological aging results in progressive attrition of telomeres, which are the repetitive DNA sequences at the end of chromosomes. These telomeres play an important role in protection of genomic DNA from end-to-end fusions. Telomere maintenance and telomere attrition-associated senescence of endothelial and smooth muscle cells have been indicated to be part of the pathogenesis of degenerative vascular diseases. This systematic review provides an overview of telomeres, telomere-associated proteins and telomerase to the formation and progression of aneurysms of the thoracic ascending aorta. A better understanding of telomere regulation in the vascular pathology might provide new therapeutic approaches. Measurements of telomere length and telomerase activity could be potential prognostic biomarkers for increased risk of death in elderly patients suffering from an aortic aneurysm.

## 1. Introduction

Telomeres are tandem DNA repeat sequences at the end of chromosomes, which maintain genomic stability during cell divisions [1,2,3]. Unfortunately, telomere length (TL) shortens with every cell replication. This results in the DNA polymerase inability to precisely replicate the set of chromosomes [4,5]. The “end replication problem” results in the critical shortening of TL or instability of the telomeric binding proteins (TBPs). This induces an arrest of cell division or a replicative senescence [6,7,8,9], which may cause genomic instability. On average, cells reach senescence after approximately 50 divisions. TL can reflect cellular turnover and is therefore used as a surrogate marker for age and as a feasible biomarker for age-related diseases. These include cardiovascular diseases and infections, which are proven to have accelerated telomere shortening [10]. TL has its potential prognostic value related to marking the specific stages of disease progression. Despite the value of maintaining TL, a limited number of cells, especially in cells with a high turnover (e.g., tumor and epithelial cells) are able to reactivate the enzyme telomerase. Telomerase, a reverse transcriptase enzyme, adds repeat sequences to the end of chromosomes [7,11]. This ensures prolonged cell survival and extended proliferation [3,5]. Moreover, telomerase presents extra-telomeric functions of enhancing cell repair mechanisms and stress resistance, as well as down-regulation of apoptotic effectors. Seen as a whole, it is clear that telomerase functions support DNA stability [12,13,14]. 

Aortic aneurysms are characterized by local inflammation with degeneration around the aorta, which leads to weakening and widening of the vessel. The formation of aortic aneurysms can be congenital or acquired and occur at different locations of the thoracic or abdominal wall. Abdominal aortic aneurysms (AAA) are strongly associated with arteriosclerosis and inflammation [15], while thoracic aortic aneurysms (TAA) are consequences of degenerative processes, hypertension or genetic mutations in rare disorders (Ehlers-Danlos or Loeys-Dietz Syndrome) [16,17]. The findings of Morgen et al. demonstrate that advancing age is associated with telomere uncapping in arteries, which is linked to senescence, which occurs independently of telomere shortening [18]. While it has been confirmed that chronic inflammation in AAA plays a crucial role of aneurysm formation, the TAA pathway still has to be discussed and requires further research.

Vascular aging involves senescence of endothelial cells (EC) and vascular smooth muscle cells (vSMC), which can be caused by telomere shortening. Several studies have shown an association of the reduction of TL in blood cells with several vascular diseases [19,20,21,22,23,24]. A major vascular disease affecting the aorta is the sporadic thoracic aortic aneurysm, which has particularly increased incidence in the elderly population [16,25,26]. 

The aim of this review is to summarize the role of TL and maintenance by telomerase in vascular diseases of the thoracic ascending aorta. We discussed the results and main conclusions that have identified shorter TL as a primary abnormality in the pathogenesis [27,28] and the influence of telomere function on the risk of vascular diseases.

## 2. Telomeres: Structure, Functions and Maintenance

The main function of telomeres is to protect the end of chromosomes during replication. This is essential for protecting the integrity of genomic material. Thus, complete replication of linear DNA molecules is not possible, which was originally proposed as the “end replication problem”, by James Watson and Alexey Olovnikov [29,30] in the early 1970s. The removal of the terminal RNA primer on the lagging strand leaves an un-replicated gap, resulting in loss of terminal sequences and genomic instability. The ability of telomeres to safeguard genomic material is enabled by the dynamic structure of telomeres and TBPs. Human telomeres contain repeats of sequence 3′-CCCTAA/5′-TTAGGG that have 2–50 kilobase pairs and a G-tail of 100–250 bases detected throughout the cell cycle [31,32,33]. However, the “Shelterin complex” contains up to six core components. These are telomere repeat binding factor 1 and 2 (TRF1 and TRF2), TRF1 interacting nuclear factor 2 (TIN2), protector of telomeres 1 (POT1), repressor/activator protein 1 (RAP1) and tripeptityl-peptidase 1(TPP1), whereby three of these factors are associated directly with the telomeric DNA either in its single stranded (ss) (POT1) or double stranded (ds) (TRF1, TRF2) form. These components play an essential role in telomere protection and telomerase regulation [33]. The Shelterin complexes selectively bind with high affinity to areas of the telomere where ss/ds-DNA junctions carry POT1 and TRF1 or TRF2, such as the 3′-telomeric end [34]. Telomeric zinc finger-associated protein (TZAP), a specific telomere-associated protein, binds to long telomeres with low concentrations of Shelterin complex by competing with TRF1 and TRF2. The TZAP binding triggers telomere trimming, which is an additional mechanism of TL control [35]. However, depletion of TRF2 induces chromosome end-to-end fusions, which result in loss of telomeric sequences and consequently leads to critical telomere shortening found in human cancer and cells [36]. 

Telomere-associated proteins influence the control of TL and DNA damage, which involves: (i) proteins acting as activators of the Wnt signaling pathway; (ii) modulating Ataxia-telangiectasia mutated kinase (ATM)-dependent Ataxia telangiectasia and Rad3-related protein (ATR), which is activated in response to damaged caused by DNA-double strand breaks (DSB) [37]; and (iii) poly-ADP ribose polymerase-1 (PARP-1), which is important for the normal or abnormal recovery of DNA damage [38]. In particular, TRF1 and TRF2 can be modified and regulated by PARP enzymes [39,40].

Telomere homeostasis involves different factors that are involved in telomere shortening or attrition and maintaining TL. A new technique developed in the Mai Laboratory in Winnipeg allows the detection of telomere anomalies (attrition, aggregates) in 3D [41,42]. Telomere shortening is posed by telomere attrition during cell division or direct DNA damage on telomeric sequences. Telomeric DNA damage can be caused by oxidative stress, loss or deficiency of TSB or degradation of RNA primers of proteins involved in DNA repair (e.g., Ku 70/80, PARP-1). In addition, homeostasis of telomeres is regulated by DNA methylation, which enables telomeric-stabilized protein inhibition or expression [41]. 

Telomerase counteracts telomere shortening to maintain telomeres in the vasculature. Still, telomere attrition occurs with each mitotic cycle due to the fact that telomerase activity (TA) is typically low or absent in human somatic cells [3,42,43]. Telomerase contains two main components to compensate this loss by adding DNA sequences to the end of chromosomes. The first component is hTERC, an RNA component, and the second hTERT, a reverse transcriptase. While hTERC expression extends telomerase reactivation, hTERT limits telomerase activity [44,45,46,47]. Further, hTERT regulates is transcriptional genes involved in cell proliferation and extension of the lifespan in numerous cell types, such as vSMCs, fibroblasts (FBs) and ECs.

The latest findings of the non-canonical functions of telomerase have shown that hTERT expression influenced cell growth, regardless of telomere maintenance [48]. Moreover, extra-telomeric features of telomerase uncovered an independent role of telomere extension [49]. In detail, the non-canonical functions of the hTERT catalytic subunit are particularly involved in cancer progression with two major signaling mechanisms, which are namely the NF-κB and Wnt/β-catenin pathways. TERT conducts as a transcriptional modulator in tumor cells to sustain its own levels and to control the induction of target genes critical for tumor cell survival and proliferation [49]. 

A second and rare maintaining mechanism of telomere length found in approximately 10–15% of tumors is a recombinant process, the alternative lengthening of telomeres (ALT), which results from homologous recombination [50,51]. The ALT mechanism has not been found in human aortic cells and has not been shown to be active in TAA or AAA. 

Telomere exhaustion is a major age-dependent problem as aging is known to be a risk factor for vascular diseases [48]. The environmental impact might also be of great importance for telomere shortening during the development of aortic diseases. Thus, for many reasons, TL and telomerase play a critical role in aneurysm formation, as they are associated with adverse lifestyle and cardiovascular diseases (CVD). 

## 3. Telomeres and Pathobiology of Aortic Aneurysms

Telomeres have a length genetically determined at birth. TL provides a potential marker for an individual’s biological age. There is a lack of knowledge about genetic predispositions in telomere maintenance during aging and its direct relation to the integrity of the aortic tissue. There is little evidence for an association between the genomic determinants of TL and the risk of AAA [52]. Balistreri and his group showed a correlation between age-dependent aneuploidy and TL of the human vascular endothelium [53]. TL was inversely correlated with age and further increased significantly with the frequency of aneuploidy of vascular ECs. 

Telomere shorting and the associated genetic instability in vasculature plays a key role in TAA etiology [54]. Recent studies can be divided into two groups: (a) measurements of TL and telomere maintenance mechanisms in diseased aortic tissue, compared to healthy aortic tissue; and (b) comparative studies of white blood cells (WBC) TL with healthy and diseased aortic tissues. In this section, the telomeric specificity of aortic tissue will be summarized.

Artery walls of large vessels consist of layers known as the tunica intima, the tunica media and the tunica adventitia. Their main cell types include ECs, vSMCs, FBs and macrophages [55]. The intimal layer is essentially comprised of ECs. These cells have substantial influence on vSMC differentiation [56] and recent studies have showed that ECs can directly influence the vSMC phenotype [57,58]. The blood vessel tone is maintained by differentiated vSMCs, which regulate the blood pressure through constriction or relaxation. ECs and vSMCs are key players in vasculogenesis, which is important for structural stability and maintenance during cellular stress or vascular damage to preserve aortic integrity.

Previously, Okuda et al. assumed that a higher rate of telomere attrition enhances the rate of senescence of endothelial cells, which further increases the predilection for vascular disease. Cell senescence has higher significance in their main focus, the distal aorta. They also analyzed the proximal aortic tissue with the following main findings: the TL of intimal tissue was age-dependent, whereas the proximal medial aortic tissue near to the ascending aorta showed no significant age-dependent telomere attrition. Local factors such as shear wall stress may influence such findings being partially representative to vascular cell senescence [59]. 

The associations between telomerase, aortic aneurysms, epidemiological and clinical variables have been investigated in different studies, although they were limited to AAA. Dimitroulis et al. suggested a protective role of telomerase against AAA formation as studies showed that patients suffering from AAA had attenuated endothelial telomerase expression compared to controls [60]. However, there are almost no studies on this subject, which is why we designed a study to evaluate TL in TAAs, which involves the use of bicuspid (BAV) and tricuspid (TAV) aortic valves in TAAs [61]. We isolated aortic SMCs and subjected them to cell biological and gene expression analyses. The obtained data indicate that aneurysmal SMCs have reduced proliferation and migration rates compared to controls. Moreover, we demonstrated shorter telomeres in aneurysms compared to controls. Further analysis showed a difference between SMCs isolated from TAAs compared to those isolated from BAV and TAV. BAV SMCs had significantly shorter telomeres, whereas TAV SMCs showed reduced metabolic activity. Our study provides evidence that TAA-associated aortic wall disintegration shows similarities in both BAV and TAV, although there are still significant differences. 

Several preclinical studies support the idea that age-related endothelial and myocyte dysfunction could contribute to the development of AAA. Bhayadia et al. investigated aortas in telomerase-deficient mice and found significantly higher expressions of stress-induced senescence markers p16(INK4a) and p19(ARF) compared to wild-type mice. This observation eliminated the possibility that loss of telomerase expression or loss of previously reported telomere-independent, non-canonical functions of telomerase [62,63] result in endothelial dysfunction of the aorta. Focusing on ECs, Bianchessi et al. aimed to define whether mitochondrial DNA (mtDNA)-transcribed long-non-coding-RNA plays a role in vascular aging. Senescent cells in aortas of old mice showed increased lncRNA expression. Transient overexpression of lncRNA was shown to induce G2/M cell accumulation and replicative senescence in ECs. In vitro analysis established that these changes are induced in ECs, but not in vSMCs [64]. Boe et al. established plasminogen activator inhibitor-1 (PAI-1) as an important determinant of vascular senescence in vivo, which is one of the major anti-fibrinolytic proteins. It is expressed in ECs and appears to play a pivotal role in vascular aging and hypertension. Telomeres and TL have been examined in aortic tissue from animals treated with PAI-1. The results showed significant induction of senescence and accelerated aging, leading to the conclusion that PAI-1 inhibition maintains TL [65]. Additionally, Thannickal et al. described that the removal of senescent cells can protect the organism against aging [66]. 

In summary, shortening of telomeres, reduced telomerase function and cellular senescence of vSMCs and ECs seems to play crucial roles in the development of TAAs. Further studies with a greater cohort of samples are necessary to understand the role of genetically-determined aortic TL.

## 4. Regulation of Telomeres and Senescence in Cells of the Aortic Wall

Given the high impact of TL in cells of the aortic wall, it is essential to understand telomere regulation in an age-related manner. Previous in vivo data have suggested that telomerase activation is pivotal for the regulation of vSMC, EC proliferation and long-term cell viability [67]. 

The age-dependent function of EC impairs angiogenesis and the development of vascular diseases [68,69]. In vivo observed EC is remarkably inert until activated to proliferate after traumatic injuries, inflammation and tumor formation [70]. 

Telomere attrition has been detected in senescent ECs, even though telomerase in these cells inhibits the onset of senescence. Telomere shortening correlates with reduced growth rate of older human ECs [71,72]. Telomerase ectopic overexpression increases the proliferation rate of human ECs and vSMCs [73,74]. Studies demonstrate that the reactivation of telomerase in telomerase-negative cells can be induced by hTERT. Contrary studies in mice lacking TA show compromised cell proliferation and reduced tumorigenesis. In detail, phosphorylation of TERT is important to activate telomerase in the nucleus during vSMC proliferation [75]. Yang et al. described a resistance of hTERT-expressing ECs to the induction of apoptosis by different conditions related to parental cells at senescence [76]. Moreover, this group reported an inverse relationship between TA and apoptosis in these cells for the first time. The telomeres were only elongated to a specific length, which allowed the cells to bypass senescence, although they did not reach TL as described for hTERT-immortalized cells. 

Another stimulus for TA in ECs is the fibroblast growth factor 2 (FGF-2). FGF-2 expression also delays the onset of senescence in aortic ECs [77]. However, sirtuin-6 (SIRT6) is highly expressed in ECs, fibroblasts, embryonic stem cells and tumor cell lines, where it protects DNA repair and telomere maintenance [78,79,80]. In this context, the finding that a depletion of SIRT6 in ECs induces a senescent phenotype suggests that increasing the levels or activity of this protein may be a relevant approach for delaying vascular ageing.

Importantly, senescence of vSMCs contributes to aging and age-related diseases of the cardiovascular system. Prolonged cell arrest in mitosis may cause disorder of TRF2 and the telomere structure [81]. Accordingly, Bielak-Zmijewska et al. analyzed the pathways of replicative senescence of vSMCs in vitro. The cellular senescence was induced in doxorubicin (DOX)-treated cardiomyocytes, which led to a stress-induced program of senescence (SIPS) in these cells as previously described [82]. A major role of telomere dysfunction is the stress-induced senescence and apoptosis [83]. Ogawa et al. demonstrated that mitogen-induced TA in vSMCs is inhibited by peroxisome proliferator activated receptor-γ (PPARγ) ligands. Furthermore, vSMC treated with PPARγ ligands reduced cell proliferation, which was prevented in cells overexpressing TERT. This finding characterizes TA as an important anti-proliferative target for PPARγ ligands [84].

Hypoxia is a well-known regulator of vascular function and structure, which extends the proliferation capacity of vSMCs through increased TA. Conversely, vSMC growth arrest occurs after telomerase inhibition [85], while senescent vSMCs are accelerated by oxidative stress-induced DNA damage, inhibition of telomerase and marked TL in arteriosclerosis [86]. Similar findings are found in ECs, where oxidative stress induces a downregulation of telomerase and increases telomere damage [87]. Antioxidants prevented TERT downregulation in ECs [88]. In atherosclerotic vessels, telomere shortening is prone to high hemodynamic stresses, which might also enhance EC turnover [59,71].

## 5. Factors Affecting Telomeres and the Risk for TAA Formation

In vitro and in vivo findings have analyzed the link between telomeric integrity and factors influencing TAA formation. First, we discuss unifying pathophysiological mechanisms, which are responsible for ageing and age-related disorders.

### 5.1. Oxidative Stress

Accumulation of oxidative damage plays an important role in age-related telomere dysfunction of vascular cells [89]. Oxidative stress is defined as an increase in the intra-cellular concentration of reactive oxygen species (ROS) [90,91]. ROS are generated during the regular mitochondrial electron transport chain. Other ROS species can be derived from superoxide and hydrogen peroxide, which are associated with the depletion of extracellular matrix proteins. ROS is proposed to be involved in TAA development. In aortic tissue, increased ROS levels were detected in aneurysmal aortas compared to non-aneurysmal aortas of patients and were confirmed to play an important factor in the development of aneurysms [92]. Knockout mice with suppressed ROS production had reduced TAA formation [93]. Moreover, ROS induces genomic instability and loss of DNA stabilization factors, which was shown in thoracic aneurysmal vSMCs [94]. 

Telomerase is not only located within the nucleus and cytoplasm, but also be found in the mitochondria [95]. The telomerase located in the nucleus plays a crucial role in telomere elongation, gene expression regulation, chromatin organization and DNA-damage responses [96]. In contrast, the telomerase found in mitochondria is involved in regulation and influences apoptosis, stress protection, transfer-RNA-dependent reverse transcriptase and RNA-dependent RNA polymerase. However, in context with ROS and oxidative stress, mitochondrial telomerase reduces the production of ROS and protects mitochondrial DNA from damage [62,63]. Interestingly, telomerase seems to have an anti-apoptotic role, with the potential to block mitochondrial [14] and death receptor pathways [97].

Telomere shortening has been associated with oxidative stress by finding oxygen radicals in EC, vSMC, epidermal cells and WBCs from AAA patients [98]. Most recently, Rehh et al. demonstrated that triplet guanines present in telomeric TTAGGG-repeats is linked to the preferential accumulation of oxidative stress-related damage in telomeres [99]. An inverse correlation was detected between TL and high levels of oxidative DNA damage in WBCs from AAA patients. This suggests that oxidative stress has systematic effects, similar to telomere shortening [97]. The relationship between telomere shortening and oxidative stress is still under discussion. 

The studies are suggesting the idea that oxidative stress inhibits TA and causes telomere attrition. Moreover, the non-canonical mitochondrial specific role of TERT is focused on protection against oxidative stress. Therefore, a regulation of TERT will be a promising target in the development of therapeutic treatments, which affecting telomerase, in aging and telomere related diseases.

Beyer et al. identified that increasing TA reverses the pathological phenotype seen in CVD [100]. This study supports the hypothesis that TA in mitochondria regulates ROS production with direct physiological impact. Moreover, new findings in CVD showed that nicotinamide adenine dinucleotide phosphate (NADPH) oxidase plays an essential role in the development of diseases. For example, Xu et al. have demonstrated that NADPH oxidase increases the proportion of SMC phenotypes associated with atherosclerotic plaques vulnerability [101]. There is a lack of data related to these findings in TAAs, although this should be a promising field of disease research.

### 5.2. Nutrition

It is well known that an increase of caloric intake and, consequently, obesity are known to reduce the average age. Specially, the adipose tissue plays a crucial role, which influences the function and structural integrity of the cardiovascular system as a source of ROS [102]. Unhealthy diet, high glucose (e.g., fructose) and low-density lipoprotein intake [103] are associated with an increased risk of CVD. As human primary cardiovascular cells show phenotypic and molecular changes. Simulated unhealthy diets showed enhanced proliferation of SMCs and increased senescence as well as loss of endothelial nitric oxide synthase (eNOS) in ECs in vitro. Further, the protein restriction during lactation for maternal animals showed a reduction of DNA damage and shortens telomeres in aortic tissue. These results are associated with reduced 8-hydroxy-2-deoxyguanosine, a marker of oxidative stress, which has a positive effect on child growth and cardiovascular diseases [104]. 

### 5.3. Hypertension

Hypertension is a significant contributor to telomere attrition [105]. The potential relationship between TL and human hypertension were examined in several studies. Two large studies, the Framingham heart study and the cardiovascular health study, have suggested that hemodynamic stress in linked to telomere shortening [24,106]. In the Framingham Heart Study, leukocytes TL of hypertensive male subjects are significant shorter compared with their normotensive peers. 

The molecular basis of the abnormality in hypertension has not been fully defined as vascular remodeling in arterial vessels with high blood pressure. Studies in telomerase-deficient mice have shown a direct association between hypertension and telomere attrition [107]. Cao et al. designed an animal study with spontaneously hypertensive rats and analyzed aortic tissue [67]. Telomerase was selectively activated, while telomeres were lengthened in vivo and in vitro. Moreover, telomerase was found to play a significant role in aortic tissue. Thus, the downregulation of hTERT arrests the increased proliferation of vSMCs in hypertensive rats. In addition, the study showed that the p53 checkpoint appears to be crucial for increased vSMC growth rate. The molecular targeting of this increased activity may thus provide a window of opportunity for preventing high blood pressure. In contrast, the study from Dimitroulis showed that differences in telomerase RNA expression between AAAs and controls were independent of other factors, such as age and hypertension [60]. Summarizing, a reduction of TL is associated with increased EC growth rate that seems to be a consequence of vascular shear stress.

### 5.4. Diabetes Mellitus

Diabetic patients are at higher risk of vascular diseases [108] as post-prandial hyperglycemia increases cardiovascular risk. Diabetes and hyperhomocysteinemia have been shown to be independent risk factors for increased progression of CVD, including TAA and AAA [109]. vSMC cultures exposed to high levels of glucose or/and homocyteine (Hcy) showed increased TA, which resulted in a proliferative response of vSMCs [109]. In contrast, Matsui et al. showed that high plasma concentrations of insulin therapy might accelerate endothelial senescence [110]. Human ECs exposed to high glucose increased senescence markers and decreased TA. Preserved TL and delayed senescence in ECs under high-glucose conditions were preserved with normal concentrations of insulin. Moreover, these effects were associated with reduced reactive oxygen species and increased nitric oxide (NO). These results may help to explain the complicated roles of insulin in CVD in the elderly [110]. Nevertheless, recent studies have claimed that diabetes has a protective role regarding the formation of abdominal aortic aneurysms. Further studies need to elucidate whether this protection is based on the effect of different anti-diabetic medications (which are known to potentially reduce oxidative stress within the vascular wall). 

## 6. Telomeres as Markers in Leukocytes

In the early 1990s [111], it was speculated that TL measured in leukocytes is a marker for increasing age. Biological age seems to be a more descriptive predictor of CVD rather than chronological age [112]. Non-functional vascular ECs provoke a transendothelial migration and adhesion of circulating leukocytes. These circulating blood leukocytes reflect the biological age of vascular walls and are associated with a reduction of TL and CVD [113,114].

Patients with AAA showed shorter leukocyte TL compared to controls [115]. The measurement of leukocyte DNA mirroring for vascular telomere content seems to be an accurate surrogate for human vascular age [114]. Additional factors (e.g., genetic and environmental) are involved in telomere shortening, suggested by an inverse correlation between TL and oxidative DNA damage in a wide spectrum of inflammatory and degenerative disorders [98]. The pathological progression of atherosclerosis in AAA is induced by an inflammatory process, which is mainly responsible for a high cellular turnover and therefore, rapid telomere shortening [116]. 

The risk factors for telomere attrition and a decrease of TA in TAA patients are significantly associated with an increased systemic inflammation, age, gender, nicotine abuse and high blood pressure [117,118]. An inverse correlation between short TL and chronic vascular diseases in a high-risk population of chronic kidney disease (CKD) patients was found [119]. Whereby, the most significant associations were found for carotid arteries interventions, TAA and AAA.

Interestingly, the group of Salonurmi analyzed TL in TAAs and demonstrated opposite results [113]. Salonurmi et al. detected TL by using the Cawthon’s quantitative (q) PCR methods, while other groups measured TL by comparing the mean terminal restriction fragments (TRF) length by using the more precise Southern Blot analysis. In all studies, several limitations need to be considered. TL varies between different chromosomes and cell types. Measurement of the mean TRF length is inexact to analyze cellular telomere attrition. As critical telomere shortening of a single chromosome does not affecting the mean TRF length. Therefore, one point of interest should be the signals from telomeric DNA damage in aortic tissue and WBCs of same patients. 

Yan et al. has demonstrated significantly shorter leukocyte TL in a Chinese population affected by aortic dissections compared to a control group without vascular diseases [20]. After adjustments for other risk factors the short leukocyte TL was associated with aortic dissection. 

Several factors have contributed to the lag of telomere measurements in patients and data interpretation. This includes technical obstacles for large-scale measurements and lack of standardization across techniques and laboratories, apart from the strong association with risk factors in patients. In clinical practice, age, gender and lifestyle also play an important role, which makes the usage of TL measurement as a biomarker difficult and controversial discussed. 

## 7. Mechanism to Preserve Telomere Length

A comprehensive health concept, including maintenance of high-intensity physical exercise and a well-balanced diet, has been suggested for preserving TL by activating telomerase even in old age [120,121]. The cardiovascular benefits of chronic exercise are found in WBCs isolated from endurance athletes. Compared to untrained individuals, endurance athletes have increased TA, higher levels of expression of telomere-stabilizing proteins and significant lower levels of cellular growth arrest and cell death markers. Although all parameters, such as heart rate, blood pressure, body mass index and lipid profile, are beneficial in athletes, the positive TBP regulation was independent. 

In the context of a healthy diet, several nutritional components have been shown to have a positive correlation with increased TA [122]. Balanced intake of marine omega-3 fatty acid lowers the rate of telomere shortening [123]. Resveratrol found in grapes, blueberry, raspberry and mulberry activates human nicotinamide phosphoribosyltransferase, sirtuin-4 and the reverse transcriptase of telomerase in human aortic SMCs. This also decreased the expression of p53 by 50% and increased TL, which delayed vascular aging [124]. A dietary supplement, Shoushen Granule, was shown to improve atherosclerotic lesions of the arterial wall by decreasing total cholesterol and low-density lipid cholesterol levels and increasing TA and TL in peripheral leukocytes and vascular cells [125]. Furthermore, rice bran, a by-product of the rice milling process, contains γ-oryzanol, tocopherols and phytosterols. Positively effects can be attributed to rice bran, as lipid-lowering and anti-inflammatory effects. The enzymatic extract of rice bran prevents telomere shortening in aorta and mononuclear cells and reduces vascular apoptosis and atherogenesis in mice. Presently, the exact mechanism by which resveratrol, Shoushen Granule or rice bran induces telomerase remains unknown; these findings suggest a beneficial effect in anti-aging processes in diseased cardiovascular cells.

TL can also be preserved by pharmaceuticals. Aspirin, angiotensin-converting-enzyme (ACE) inhibitors and statin therapy have been shown to have an anti-senescence effect on the vascular endothelium [126,127,128,129,130]. Pioglitazone, a drug of the thiazolidinedione class, increase TA in aortic cells and prevents stress-induced cell death of ECs. It reduces levels of senescence markers p16, cell-cycle checkpoint kinase 2 and p53 [84,131]. Activation of liver X receptors (LXRs), which are nuclear receptors, are triggers for protection of arteries against atherosclerosis. LXR ligands stabilize low levels of senescence-associated β-gal activity, and reverse the decrease of telomerase expression in human ECs. This effect of LXR activation was associated with reduced ROS and increased eNOS activity [132].

## 8. Conclusions

**Figure 1:**
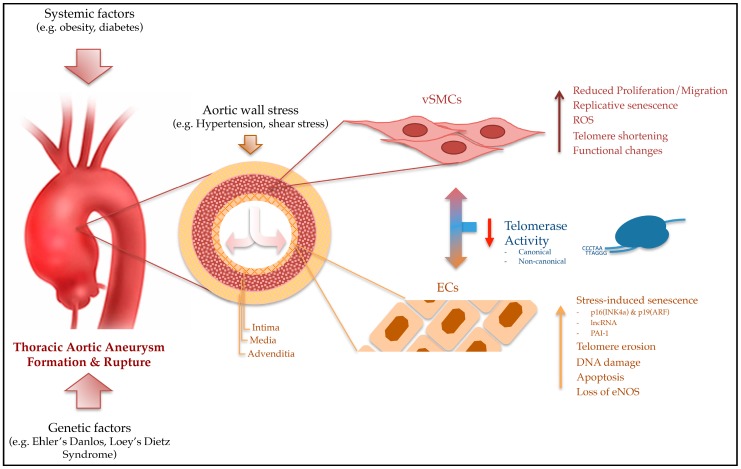
Multiple factors are associated with thoracic aortic aneurysm formation. The influence of telomerase activity on vascular smooth muscle cells (vSMC) and endothelial cells (EC) and the regulatory interaction between both cells is of particular importance.

Genetic dispositions, such as the Ehlers-Danlos Syndrome or Marfan Syndrome, and vascular risk factors (e.g., smoking and obesity) appear to be strong risk factors for AAA. Studies in animal and human trials associate a reduced TL and an inherent TA with DNA damage and increased predisposition to form AAAs. In summary, following findings show the link between telomere biology and TAA (Figure 1). (1) Shortened telomeres reduced telomerase function. Therefore, premature senescence of vSMCs and ECs are strongly associated with the development of aortic aneurysms; (2) Aneurysm formation results from abnormal proliferation and/or degeneration of vSMCs and ECs in the aortic wall, whereas the induction of TA is essential for the regulation of vSMC and ECs proliferation, which occurs independently of progressive telomere shortening; (3) Shortened telomeres in aneurysmal aortic tissue were correlated with telomere attrition in white blood cells.

A considerable number of reports suggest that telomerase and its key components, TERT and TERC, have functions beyond their ability to lengthen telomeres. TA is found in a wide range of cell types and tissue to be involved in a variety of activities, especially with non-canonical functions that have not been fully characterized. In aortic cells, telomerase seems to works as a transcription co-factor that regulates the expression of genes involved in the regulation of cell proliferation, differentiation and apoptosis. 

TL seems to be an attractive new biomarker for biological age or for follow-up of aneurysm progression over years. However, TL needs to be interpreted with caution due to the formation and the progression of TAA over decades. Little is known about telomere attrition in the earlier stages of disease. Interestingly, telomere shortening is accelerated prior to the onset of clinical disease. A variety of risk factors, such as age, gender, smoking and obesity, have a great influence on both TL and the health of most patients. Therefore, the non-specific modulations of TL suggest a poor specificity and limited potential as a biomarker of a specific disease.

Hence, the main questions related to telomere regulation and the development of aortic aneurysms remain unanswered: Does telomere-shortening lead to the development of TAA or does the DNA damage caused by external factors (e.g., hypertonic blood flow) reduce TL in aortic cells? Prospective longitudinal studies would be required for accurate delineation of true changes in TL.

## Abbreviations

AAA	Abdominal aortic aneurysm
ALT	Alternative lengthening of telomeres
BAV	Bicuspid aortic valve
CKD	Chronic kidney disease
CVD	Cardiovascular disease
DOX	Doxorubicin
ds/ss	Double-stranded/single-stranded
DSB	Double strand break
EC	Endothelial cell
eNOS	Endothelial nitrate oxide synthase
FB	Fibroblast
LXR	Liver X receptor
NAMPT	Nicotinamide phosphoribosyltransferase
NO	Nitride oxide
PAI-1	Plasminogen activator inhibitor-1
PARP	poly-ADP ribose polymerase
POT1	Protection of telomeres 1
RAP1	Repressor activator protein-1
ROS	Reactive oxygen species
RTL	Relative telomere length
SIPS	Stress-induced senescence program
(v)SMC	(vascular)smooth muscle cell
TA	Telomerase activity
TAA	Thoracic aortic aneurysm
TAV	Tricuspid aortic valve
TBP	Telomere binding protein
(h)TERC/(h)TR	(human)telomerase RNA-component
(h)TERT	(human)telomerase reverse transcriptase
TIN2	TRF1-interacting protein 2
TL	Telomere length
TPP1	Tripeptidyl peptidase 1
TRF	Telomere repeat-binding factor
TZAP	Telomeric zinc finger-associated protein
WBC	White blood cell
